# The First Prescription

**Published:** 1895-01

**Authors:** Olin M. Drake

**Affiliations:** Boston, Mass.


### THE FIRST PRESCRIPTION;

### OR, THE ESSENTIAL FACTORS TO BE CONSIDERED IN MAKING A HOMŒOPATHIC PRESCRIPTION.*

OLIN M. DRAKE, M. D., BOSTON, MASS.

*Gentlemen of the Bœnninghausen Club:*—First allow me to thank you for your flattering invitation to write upon so difficult and important a subject as the above. The treatment of this subject is, I fear, beyond my limited abilities. However, I shall attempt to realize some of your expectations.

But before entering into the analytical study I have undertaken I wish to proclaim my medical creed, which, I believe, is similar to yours. I accept all of Hahnemann's doctrines, without any reserve whatever. I recognize that there is but one universal law of cure, and that is the similar; that all diseases are due to potential and dynamic alteration of health; that all medicines produce physiological and dynamic effects identical with the manifestation of disease; and, finally, that the successful application of this divine law of cure depends entirely upon the similarity between the medicinal and natural diseases.

In all systems of science or medicine certain rules must be followed, in order to establish the reliability or unreliability of its principles. The rule laid down by the founder of Homœopathy, will bear the crucial test, as we all know. While the guiding principles formulated by our master are ever the same, yet their application varies according to the symptoms of each patient, and hence the difficulties in their practical enforcement. But when they are fully understood and faithfully applied, grand

---

* Read before the Bœnninghausen Club, December 12th, 1894.

and brilliant results are inevitably secured, demonstrating perfectly the truth of the law of similars.

Before beginning my comments upon these remarkable doctrines it will be better to say a few words about Hahnemann's classification of diseases. He divides diseases into acute and chronic. The acute originate from defective hygiene, moral and mental, telluric or meteoric influences, but they may also be due to a recrudescence of a latent psora; while the chronic arise from three miasms: psoric, syphilitic, and sycotic (*Organon*, pars. 73, 74). Hahnemann does not classify among the chronic diseases patients who suffer from living under baleful or unhygienic influences, or are guilty of excesses, or undergo privations, or are exposed to trying mental conditions, providing no chronic miasm exists (*Organon*, par. 77); but he considers as belonging to that category the many victims of prolonged allopathic heroic medication (*Organon*, par. 75).

The above classification shows the great importance Hahnemann attached to our discovering the provoking cause of every case of disease investigated. He urges us, besides, to collect all and every detail relating to the case, and afterward to consider the *ensemble* of the symptoms. His directions in relation to the examination of the patient (*Organon*, pars. 83 to 104) have never been equaled before or since his day, and stamps him as a nosologist without a peer. Undoubtedly, Hahnemann's method of collecting all the symptoms, with their distinctive features, including the conditions which aggravate and ameliorate, with the accessories, etc., is the only true one upon which to found a diagnosis, as well as to recognize the actual morbid peculiarities of the individual. Ætiology and symptomatology are thus the main reliance of the homœopathician. With Hahnemann the diagnosis of a case was an object and not an end—simply a means to secure a satisfactory therapeutical solution. And, as he so ably points out, the symptoms presented by the patient are but the outward reflection of some peculiar internal modification in the functions of the body or diseased organs.

Hahnemann formulates three rules relative to prescribing:

The proper taking of the case; the selection of the simillimum; and the dose and its repetition. It is my intention this evening to treat of the second rule only, although, with your permission, I shall say a word or two about "the taking of the case." In the estimation of some prescribers this is supposed to be an easy matter; but, gentlemen, although I have been practicing Homœopathy for twenty-five years, I must frankly confess that I often find it very laborious and not seldom perplexing. Dr. Carroll Dunham says in his *Lectures on Materia Medica* (Vol. II, p. 42): "It is the most difficult part of our duty." And Dr. P. P. Wells tells us in his work on *Intermittent Fever:* "It took me thirty years to learn how to examine a case for prescription." Now, when such generally acknowledged able physicians speak thus on this subject, it stands to reason that I, of inferior mould and skill, should frequently find it puzzling. And does not Hahnemann tell us in his *Organon* (par. 104) that when we have taken the case accurately "the most difficult part (of our work) is accomplished"?

The treatment of acute diseases is often, comparatively speaking, easy, for the symptoms develop rapidly and are generally more accentuated, and, besides being recent, are fresh in the minds of patient and attendants. We thus more easily get at the true picture of the disease. In the case of chronic affections, however, perhaps of many years' standing, the task is much more difficult; many more things have to be looked into and considered, as we shall see later on, apart from the fact that the patient may have forgotten many of his symptoms. In summing up the value of symptoms, with the view of prescribing, some of them, among the fundamental and the secondary (individual), are of greater value in the selection of the remedy than others. Generally, the symptoms which, from a diagnostic point of view, are essential, are of little account from a therapeutical, and *vice versa.* In other words, the accessory or secondary symptoms, which are of no consequence to the pathologist, are often the main dependence of the seeker after the simillimum. Diagnostic or pathological symptoms are common to all cases of the same affection, but that which individualizes each case forms the true indication for the appropriate remedy. The striking and characteristic symptoms of the disease must be as similar as possible to the striking and characteristic of the medicine; and if the secondary symptoms also correspond to those of the drug, so much more reliable is the choice. Hahnemann says: "We ought to be particularly and almost exclusively attentive to the symptoms that are striking, singular, extraordinary, and peculiar (characteristic); for it is to these latter that similar symptoms, from among those created by the medicine, ought to correspond, in order to constitute it the remedy most suitable to the case. On the other hand, the more vague and general symptoms, such as loss of appetite, headache, weakness, disturbed sleep, uncomfortableness, etc., merit little attention, because almost all diseases and medicines produce something of such general nature." (*Organon*, par. 153.)

To illustrate the value of these directions of Hahnemann, let me submit to you a case which, from this particular point of view, must interest you all: In October, 1876, there came under my care a hard-working man of fifty-five years. The preceding Fourth of July, while working in the hot sun, bare-headed, he received a sunstroke. In a semi-conscious condition he was conveyed to his home, and received there the attention of an allopathic physician up to the time of my first visit. I am unable to give you his symptoms, for I cannot find my record-book of that year; but I remember that he was a wreck, mentally and physically. His condition was such that I could give his family no special encouragement, but I was, nevertheless, extremely anxious to help him, and assumed charge of his case. According to the totality of his symptoms, Lycopodium was the remedy indicated, and, after careful study, I decided to prescribe it in the 200th (Dunham). For a number of weeks its action was most satisfactory. The patient presently was able to go about among his neighbors, but was, however, unfit for physical work. I studied and studied his symptoms, and yet I could not find a better indicated remedy than Lycopodium. I gave it very high, and I gave it very low, but there was no favorable response. Sulphur$^{200}$ was next given, and then Lycopodium was again tried; still there was no reaction. Knowing full well that the difficulty was not with Homœopathy, but with my own ignorance, I had that man come to my office several times, and I would note down his symptoms anew each time, as though it were the first consultation. And yet I could make nothing out of the *ensemble* of the symptoms but Lycopodium. One day, while questioning the patient, I noticed that he repeatedly passed his hand downward from the forehead over the nose, as if to brush something off. I asked him why he did this, and he smilingly answered that for a long time he had had a feeling over the bridge of his nose, as though a horse-hair was drawn tightly across it, and every little while he found himself trying to remove it. He also told me that occasionally he had the sensation of having on spectacles with bows pressing unpleasantly upon the back of the ears. This feeling he was also unable to brush away. These indications led me to the study of Tartar-emetic—a remedy ranking very low in summing up the totality of the symptoms; but it had in its pathogenesis the more marked and peculiar symptoms, and several others. I gave it in the 200th (Dunham), and within twenty-one days my patient was perfectly well and able to return to his work again.

Can any of you gentlemen tell me the significance, from a pathological point of view, of this particular "striking, peculiar and uncommon (characteristic) sign and symptom"? and yet, as we all know, it is often just such symptoms, trivial as they may seem, which guide the homœpathician to the true curative remedy.

I am tempted to give you another case. In 1871, I was consulted for one of the worst cases of chlorosis that I have ever treated. A girl of sixteen, living in Charlestown, Mass., was for three years under the care of two noted allopathic physicians of this city, but steadily growing worse from day to day. At this stage of her illness her parents removed her to her former home, in Maine, and then the patient came under my care. On account of lack of space, I shall mention only her most marked symptoms, which I considered the key-notes. She had a mania for eating pins and needles; she would spend much of her time in playing with and rolling them in her hands or fingers, forming fantastic figures with them, by sticking them into some fabric, etc., and, finally, she took to swallowing them. When I tell you, gentlemen, that this girl swallowed hundreds of pins and needles, I do not exaggerate. She knew well that she ought not to make a diet of such food, but if she saw a pin or needle on the floor, she would stand as rigid as a marble statue with her hands clasped behind her back and begin exclaiming, " Pin, pin," or " Needle, needle." If some member of the family did not " hustle about pretty lively " and seize that pin or needle, she would swoop down upon it, like a shanghai fowl on a bug. In looking for medicines having in their pathogeneses symptoms pertaining to pins and needles, I found in the old work of Jahr's and Possart's *New Manual*, under Silicea, " fixed ideas about pins," and in Jahr's *Mental Diseases*, " Monomaniacal ideas about pins, which she sees everywhere and dreads." The similarity between this and my patient's mental condition seemed somewhat vague, but Silicea appeared well indicated otherwise, and as the mental symptoms were the last to appear, I gave Silicea$^{200}$ (Dunham). The improvement was not one marked for its rapidity, but was gradual. The fondness for pins and needles was the first symptom to disappear, and the others followed in the inverse order of their appearance. Six months later my patient was well, and is well to-day. She is now the mother of three children.

Of almost as great importance as the extraordinary and peculiar symptoms in deciding the choice of a remedy are the more recent evolutions of symptoms of disease. These later symptoms must be considered or the simillimum will escape the prescriber. This Hahnemann dwells upon frequently in his writings, and in practice all homœopathicians daily realize its supreme value. To my knowlege of this fact I am indebted for the performance of cures which otherwise could not have been accomplished.

The complicated and confusing picture offered by a patient who has been using drugs is often a difficulty in the way of accurate prescribing, rendering successful treatment very difficult and not seldom impossible. We must, under such conditions, ascertain which were the symptoms present before any medicine was taken, and which followed its use. The former and the symptoms that appear several days after the discontinuance of the medicine, give the true and original picture of the case (*Organon*, par. 91), and should be the main factors in deciding upon the remedy. Many of us, when called upon to prescribe for a patient who has taken many drugs, are in the habit of giving Nux-vomica as the first prescription. Now, is this Homœopathy? We all can recall cases in which this remedy has worked marvels, and as I write I am reminded of one which I will relate.

I was requested, some time in 1871, to visit a man of about forty-five years, who had been ill for some fifteen months. Three physicians, two allopathic and one eclectic, had stated that he was suffering from cancer of the stomach. Of course, he had been given up as incurable. I can see him at this moment, seated upon the side of his bed, supported by his wife on one side and his daughter on the other; between his feet was a wooden bucket, into which he had vomited some two quarts of a very brownish or almost black fluid, with slime and particles of food taken the night before. His skin was extremely dry and rough, of a marked yellowish hue; his bowels had been in a torpid state for months, and his urine very scanty. As to his physical appearance, he was what I would call *skeletonized.* I never saw anything like it before or since. If you placed him upon his back in bed his spinal column formed a ridge up the abdomen; in fact, he was so thin that, for the life of him he could not tell whether his pain was in his back or belly. This emaciation was general, save his feet and ankles, which were œdematous. In the epigastrium was a tumor the size of an egg, which could be easily taken between the fingers, having a hard, nodular feel, and seemed to be situated about the middle of the greater curvature of the stomach. He had been in good health all his life previously, with the exception of salt rheum. This had been a source of much suffering to him until about two years before, when it was removed by an ointment. Shortly afterward he became ill, and then began a most severe course of drugging. Without giving his symptoms special study, I left him Nux-vomica $^{200}$ (Dunham), which he took for forty-eight hours, at intervals of two hours. On the occasion of my next visit I found him so much better that I discontinued the remedy. Two days later I found him still improving, no vomiting, sleep better, no distress at the stomach, and the bowels had moved, without aid, for the first time for months. He was now, however, complaining of a tremendous itching all over him, without any eruption—a pruritus. I continued the Sac-lac. In a few days he was "a sight to behold;" he reminded me of a case of confluent small-pox, though there were no pustules, but he was literally covered with a moist, eczematous eruption, and the itching and burning were dreadful. The man actually wept when I would not allow him to use any external application. To make a long story short, the patient began to improve soon after. The Nux was allowed to act for six or seven weeks longer, when I repeated it in the 50 M (Fincke), one dose. Some time later my patient had an attack of piles, for which I prescribed Sulphur $^{200}$ (Dunham), and this was the last medicine he required. His recovery was complete. I saw him thirteen months ago. He told me that he had never been ill since, and was then weighing about two hundred pounds.

Hering tells us: "If our patient has been drugged by the old school, we must direct our antidotes principally against the last given drugs. For instance, against abuse of Alcohol or aromatics, Nux-vomica; against Tea, Pulsatilla or Thuja; against Quinine, Pulsatilla, etc.; against Iodium and Iodide of Potassium, Hepar; against blistering, Camphor; against cauterizing with Nitrate of Silver, Natrum-mur.; against bleeding, purging or losses of blood, China; against mechanical injuries, by stretching, Rhus; by bruising, Arnica, etc.; against Chloroform, Hyoscyamus, etc."

Lippe also says in his *Materia Medica*, that Nux-vomica is often suitable to begin the treatment of cases after drugging; and Raue advises it also. I acknowledge the high sources from which such advice comes; but is it Hahnemannian Homœopathy for us to indiscriminately give Nux-vomica after drugging, Pulsatilla after Quinine, etc.? I think not. In relation to this matter Hahnemann pointedly says (*Organon*, par. 91): "The symptoms which appear, and the sensations of the patient during the use of medicines, or shortly after, do not furnish a true image of the disease;" and, "when the disease is of a chronic nature, and the patient has already made use of remedies, he may be allowed to remain some days without giving him any medicine;" but, advises us (*Organon*, par. 92), "when an acute disease is to be treated, so dangerous in its nature as not to admit of delay, and the physician can learn nothing of the symptoms that manifested themselves previous to the remedies, then he (the physician) is to view the whole of the existing symptoms—including in one and the same image the primitive disease and the medicinal affection conjointly," and then choose the simillimum from the *ensemble* of the symptoms. I believe that Hahnemann's advice in the long run will prove more satisfactory, as a strict adherence to all his rules inevitably does. Hahnemann gives further instructions on this topic in his *Chronic Diseases*, pp. 145 to 148.

This subject has been recently discussed by several members of our school. They contend that the drugs which have been administered too freely to the patient should be corrected by the administration of the same remedy, in a high potency. We are promised an article from the pen of an esteemed friend and colleague, E. W. Sawyer, M. D., of Chicago, on this subject, setting forth his theory and experiences; and I am interestedly looking forward to its appearance. My views may be changed by this paper, but I have my doubts about it at present.

When we suspect the existence of chronic miasms, we must study the symptoms with the greatest care. We must endeavor to discover which miasm exists, and make sure whether two or more do not prevail (*Organon*, par. 206). The keenest judgment is necessary in all our examinations, but more especially in the case of specific diseases, for some patients will conceal the very facts we must know. When we have discovered the particular miasm under investigation, we must next ascertain what treatment he has received (*Organon*, par. 207). If any medicine has altered the course of the disease, or added symptoms of its own to the case, we must know it, so as to resort to the proper antidote (*Organon*, par. 207). In the *Chronic Diseases*, Vol. I, p. 113, Hahnemann directs, in relation to this subject: "First we annihilate the psoric miasm by the subsequently indicated antipsorics; then we use the remedies indicated for sycosis, and lastly, those for syphilis. These different orders of remedies are alternately employed, if necessary, until the cure is completed." Once all the facts bearing upon the case in our possession, we then begin the study of the *ensemble* of the symptoms, endeavoring to recognize the particular symptoms that individualize that particular case. The apparent contradictory statements in the *Organon*, par. 18, where we are told by Hahnemann that we must take the totality of the symptoms, and paragraph 153, wherein we are instructed to single out the characteristic indications, puzzled me in my earlier years of practice, as I know it has many of my colleagues. I have, however, since seen my mistake. To any one who will reflect seriously over the matter, there is actually no contradiction in these two paragraphs. Hahnemann taught us to weigh carefully every symptom with its conditions, accessories, etc., for these are our only and true guides in searching for the appropriate remedy; but all symptoms are not of equal value, as has been already stated. Some symptoms are characteristic and determining, while others are general and belong to many medicines. Thus it will be seen that Hahnemann did not content himself with treating simply a series of symptoms, without any further thought, as his detractors charge him with. There is no other way of finding the curative remedy than by the study of the entire group of symptoms, with the qualifications just referred to. This method Hahnemann has repeatedly promulgated in his writings and in his advice to his pupils, and it forms in truth at once the great difficulty and the great strength of homœopathic therapeutics. The secret and the difficulty of the Hahnemannian method thus consists in knowing how to individualize a morbid state, and laying particular stress upon the guiding symptoms.

This matter of the relative value of symptoms is one of the greatest moment, and the skill of the homœopathician in differentiating between the essential and non-essential symptoms, makes the successful or unsuccessful practitioner. To put it differently, the physicians who generalize fail, while those who individualize succeed. Both pathological and pathogenetic knowledge are necessary to enable one to make the right selection of the remedy.

Before dismissing entirely the supposed inconsistency of Hahnemann, above referred to, let me say to those who wish for further enlightenment on this topic, to peruse the following very clever articles: HOMŒOPATHIC PHYSICIAN, Vol. I, p. 15; *Trans. of the I. H. A.* for 1888, p. 65, and *The Organon*, Vol. III, p. 435.

In some instances, as in the so-called one-sided diseases, we may be unable to find a remedy which will cover the totality of the symptoms of the disease, owing to the imperfect proving of some of the remedies in use, or to the fact that the required remedy has not been incorporated in our materia medica. We are then taught to select the remedy that most clearly resembles the symptoms present (*Organon*, par. 162). From the use of such a remedy we must only expect a partially curative effect, and often during its administration, or following it, symptoms will appear that were not present before. These Hahnemann calls accessory symptoms, and are the outcome of the employment of a similar instead of a simillimum. Their appearance are of no detriment to the patient (*Organon*, par. 164). The remedy administered will have this further effect, if properly chosen, of removing some of the morbid symptoms—those similar to it. And this constitutes the first step in the direction of a cure (*Organon*, par. 163). But, on the other hand, if among the symptoms of the remedy given, not one bears a perfect resemblance to the salient and characteristic symptoms of the disease, we cannot expect from it immediate beneficial results (*Organon*, par. 165). Fortunately for us and our patients, such cases are rare, says Hahnemann. The closest similar will cause an alleviation in the original symptoms, and besides, subsequently, new and more characteristic ones will likely appear, which will facilitate our next selection (*Organon*, par. 166).

If the accessory symptoms caused by the remedy (*Organon*, par. 163) are of any gravity, we do not allow the effect of the remedy to exhaust itself; we re-examine the patient and prescribe upon the new picture of the disease (*Organon*, par. 167), upon what Bœnninghausen calls the new "symptom-complex." The simillimum is now found more easily from among the better known and analogous remedies. If the new remedy then selected should not be sufficient to completely cure, we examine the remaining symptoms of the patient and select the remedy which is most appropriate to the new image found. We must continue this process until our patient is completely restored to health (*Organon*, par. 168). I am inclined to believe that this is what the late lamented Dr. Ad. Lippe meant when he said that there were cases where it seemed impossible to find the simillimum, and then we had to "zigzag" our patient back into health.

Again, it not unfrequently occurs, that two related medicines are about equally indicated—the one homœopathic to one part of the disease and the second to another. This is often due, of course, to the poverty of our materia medica. It is then by no means allowable, after using one remedy, to take the second without making a new examination, because the medicine originally under investigation may not, under the actual conditions, be proper for the remaining symptoms. In that event, a new simillimum must be selected, based upon the changed state (*Organon*, par. 169). If, however, after the re-examination of the existing symptoms—without reference to the second medicine in our first study, it should still prove to be the most homœopathic, it should certainly be given (*Organon*, par. 170).

In non-venereal chronic diseases, of psoric origin, consequently, we are often obliged to employ several remedies, one after the other. Of course each must be chosen homœopathically to the group of symptoms still existing, after the preceding remedy has exhausted its action (*Organon*, par. 171). Related remedies must be selected in preference to others; this is a matter of no little importance and should not be overlooked.

The fewness of symptoms in affections classified as partial, on account of the limited number of striking symptoms, often render their treatment uncertain and difficult. It is only chronic diseases which offer this feature or difficulty (*Organon*, pars. 172, 173). The only manifestation or evidence of disease in these states is, perhaps, either an internal malady, such as headache, obstinate diarrhœa, or chronic cardialgia, etc., of long duration, or an external lesion. If due to the latter, they are called local diseases (*Organon*, par. 174). In the partial diseases of an internal origin, Hahnemann reminds us that the paucity of symptoms obtained may often be owing to the physician himself, who is inattentive or not observant enough to find more (*Organon*, par. 175). There are, however, some few diseases which offer but one or two marked symptoms, the others being barely noticeable (*Organon*, par. 176). When this does occur, which is seldom, we begin the treatment by selecting a remedy which covers those few symptoms (*Organon*, par. 177). If the carefully selected remedy should then happen to be similar to the latent symptoms, as well as the outward, visible ones, as is often the case, a cure must follow, since the strictly homœopathic remedy has been secured. It is more likely, however, that the medicine chosen is but partially the simillimum, since we had so few symptoms upon which to base a prescription (*Organon*, par. 179). Under such circumstances we may expect to see arise accessory, analogous symptoms, as in the case when the choice was imperfect (*Organon*, par. 162). These symptoms may not only belong to the remedy itself, but they may, in truth, have been already present, without the patient being aware of their existence. The medicine which has the inherent power of causing them, simply determined their appearance, or removed them, if already present, but unnoticed. For this reason these symptoms have to be considered as a part of the picture of the case. It must be remembered, too, that they may be due to dietetic errors, excitement, interference with menstrual functions, pregnancy, etc. (*Organon*, par. 181). When these symptoms have abated, should the partial symptoms be improved we know all is well with our patient; if not, a new remedy must be sought for.

Should the violence of these recently developed symptoms necessitate immediate interference, which must be seldom, owing to our minute doses, more particularly in chronic diseases, it is necessary, when the first remedy has not been followed by satisfactory results, to draw another picture of the disease, upon which to select a second, more suitable medicine. This selection will be the easier, that the number of symptoms is larger and more complete (*Organon*, par. 183). Occasionally, owing to the torpor of the nervous system, the patient may not clearly perceive, or be able to define his symptoms, although he feels very ill, then Opium (high) will remove this state, and the symptoms of the disease will appear more clearly. After the complete action of our second prescription, we note the remaining symptoms, and the picture obtained forms the guide for a third. This process is maintained until a cure is effected (*Organon*, par. 184).

The changes and sufferings, appearing on the external parts of the body, have been absurdly attributed to local causes, the general system supposedly not participating in the trouble (*Organon*, par. 185). Recent lesions, arising entirely from external injuries, are the only ones which appear to deserve this appellation. The lesion must then be quite slight, or otherwise the entire system would sympathize and fever set in, when dynamic treatment would be called for. "The reduction of dislocations, uniting wounds, extracting foreign substances that have penetrated the living parts; opening the cavity of the abdomen, either to remove a substance that is burdensome to the system, or to give vent to effusions and collections of liquids; placing in apposition the extremities of a fractured bone, and consolidation of the fracture by means of an appropriate bandage," etc., truly belong to the domain of surgery; but even then homœopathic remedies are required to aid the cure (*Organon*, par. 186).

In so-called partial diseases, not induced by physical or chemical means, or perhaps from slight external violence, the trouble undoubtedly originates internally. It would be foolish, as well as hazardous, to treat this condition as entirely local, as is the case with the old school (*Organon*, par. 187). The treatment should be directed to the cure of the disturbed vital forces; in fact, by remedies which are capable of inducing the phenomena of a general reaction in the diseased sphere. This is the only rational, safe, and radical method, fully established by repeated experiences (*Organon*, pars. 188, 189, and 190); and, simultaneously, the local and general state will disappear. And remember this happens without the aid of external applications (*Organon*, par. 191). In seeking for the simillimum here, the local as well as the general symptoms have to be considered (*Organon*, par. 192). The above remarks apply to acute local inflammation, erysipelas, leucorrhœal and gonorrhœal discharges, etc.; but if these troubles continue, after the use of the ordinary non-psoric remedies, psora is present and has to be combatted by its natural antidotes (*Organon*, par. 194). In fine, all local treatment in so-called local diseases should be totally rejected, and more particularly so if there exist any psoric, syphilitic, or sycotic miasms.

The simultaneous use of a remedy, internally and externally, in diseases whose principal symptom is a fixed local evil, offers this further objection, and a graver one, that the external trouble will disappear sooner than the internal, and cause the physician to believe that a cure has been effected, whereas it makes it more difficult and sometimes even impossible. The suppression of the local symptoms will obscure the morbific picture, and thus confuse the physician in the selection of the necessary remedy to the complete restoration of health. The principal symptoms, the local condition having disappeared, there remains only less constant and distinctive symptoms which are hardly characteristic enough to supply a clear and accurate state of the disease (*Organon*, par. 198). Its presence would also have indicated the time necessary for the complete annihilation of the disease, and its absence must necessarily prove a great drawback to the discovery of the suitable remedy (*Organon*, par. 199).

If, after the administration of the apparently appropriate remedy, the local state persisted, it would indicate that the cure was not complete; while, on the other hand, its disappearance under such conditions would conclusively establish that the disease had been thoroughly eradicated (*Organon*, par. 200).

The local malady is always a part of the general disease; in this way the vital power relieves itself, but the internal difficulty persists; in fact, gradually, in its further attempts to throw off the internal incubus, the external manifestations spread and become aggravated. In this way, we see old ulcers extend in surface, and chancres likewise, and continue to extend until the internal miasm has been corrected and removed by medicines (*Organon*, par. 201).

Any external treatment of a local symptom, whose aim is to cause its disappearance, will thus inevitably be fraught with evil consequences to the system, giving fresh impetus to the internal trouble, which had been meanwhile dormant. It is further, the source of numberless chronic affections (*Organon*, pars. 202 and 203), as we all but too well have reason to know.

As has been stated in the early part of this monograph, all chronic diseases not the outcome of defective hygienic surroundings or abuse of powerful drugs, are due to three miasms, syphilis, sycosis, and more particularly, psora. The symptoms pertaining to psora and syphilis are very ably presented by Hahnemann, but as there was comparatively little known about sycosis during his time, he touches only lightly upon this miasm. He reminds us, not to expect to find in a single patient more than a limited number of the symptoms belonging to the miasm under examination. For this reason, he advises us to complete the picture of the case, to seek for the symptoms which previously existed, and also to look into the hereditary history, including the diseases of the parents, and even grandparents. In this connection it may be well, also, to remember that the miasms of syphilis and sycosis will inevitably arouse and develop any pre-existing taint. Thus, ultimately, there may be present a combined group of the different miasms, psora, with syphilis or sycosis, and even all three together, not only defying any classification, but also greatly puzzling the medical attendant. In the event of there being present but one miasm, the knowledge that each miasm preferentially elects domicile in certain organs and tissues rather than others, assists in the diagnosis.

I believe that it cannot be gainsaid that the chronic miasms have a period of incubation within the system, previous to the appearance of the local symptoms, and all homœopaths are aware that if their local manifestations are suppressed, evidences of syphilis, sycosis, or psora must appear, sooner or later (*Organon*, par. 204). The primitive symptoms of all chronic miasms, or their secondary manifestations, for that matter, should never be treated topically, whether dynamically or mechanically. If patients did not so frequently resort to old-school measures for the removal of their local symptoms, members of our school would not have such a large class of victims requiring treatment for secondary and tertiary symptoms (*Organon*, par. 205).

Tradition and practice establish that two natural diseases which are similar or analogous in character cannot exist at the same time in the human economy without one, the weaker, being overcome and destroyed. As Hahnemann says, "Two dissimilar diseases may co-exist in the body, because their dissimilitude would allow of their occupying two distinct regions. But (in the case of similars) the stronger disease which makes its appearance exercises an influence upon the same parts as the old one, and even throws itself in preference upon those which have till now been attacked by the latter, so that the old disease, finding no other organ to act upon, is necessarily extinguished" (*Organon*, par. 45).

The experiments of Burnett with Bacillinum in phthisis and ringworm, those of Swan in the treatment of varied diseases, with their several morbid products, the successful administration of Psorinum in some cases of psora, the power of Pyrogen in controlling some of the phases of septicæmia, the good effects of Medorrhinum in some forms of sycosis, the multitude of chronic diseases which have disappeared after the patient had contracted small-pox, scarlet fever, measles, etc., etc., are all instances in point and in support of this theory. It is not impossible that we are on the threshold of marvelous developments of this method, but the homœopathician cannot go far astray who remembers the golden rule that the curative effects of medicines in disease depend upon their similarity.

The new fads of the physico-chemical school—Isopathy—notably Pasteur's rabies remedy, Koch's Tuberculin, Roux's Anti-toxine will result in incalculable mischief and misery to thousands; but Hahnemann's dynamic theory may yet dawn upon these benighted gentlemen, when a new era may arise, fruitful of much and lasting benefit to the whole human race.

Our master attached great importance to the mental symptoms, for as we have all often conclusively established in practice, they decide the choice of the remedy (*Organon*, par. 211). No thorough homœopathic cure is possible, without simultaneously considering the mental condition as well as the bodily ills (*Organon*, par. 213).

You have all noted what keen and accurate remarks are embodied in Hahnemann's observations on this subject. He tells us, "Aconite seldom or never effects a rapid or permanent cure when the temper of the patient is quiet and even; or Nux-vomica, when the disposition is mild and phlegmatic; or Pulsatilla, when it is lively, serene, or obstinate; or Ignatia, when the mind is unchangeable and little susceptible of either fear or grief."

When, in the course of disease, the ordinary calm and tranquil condition of the patient's mind is suddenly changed, under the influence of fear, sorrow, alcoholic liquors, etc., into madness or violence, thus taking on the character of an acute malady, we must resort to the non-antipsoric remedies, even if we know an internal psora is the cause (*Organon*, par. 221). Medicines of that class will lull back the patient to his former condition, when anti-psoric remedies must be used (*Organon*, par. 222), including moral and intellectual means, suitable to the nature of the case, and the best hygienic, moral, and mental regimen (*Organon*, pars. 223 and 230). In certain forms of mental aberrations, the result of perverse education or excessive indulgence of the passions, different methods may have to be put in force.

Hahnemann's advice as to the general course to be pursued in the case of demented people is characterized by a wisdom and humanity never known before his day, and only recently recognized and put in force by alienists of the old school. This alone would entitle him to the gratitude of the whole world.

Intermittent diseases, returning at fixed periods, and also those which, in certain morbid states, alternate with others of an opposite character, and at indefinite intervals (alternate species), belong to the series of chronic diseases. Most of them result from a development of psora, and occasionally, in addition, they are complicated with a syphilitic miasm. For this reason they should be treated in the first instance by anti-psoric remedies, and in the second by anti-psorics, alternately with anti-syphilitics. The required remedy must be selected from among those whose pathogenetic effects are similar to the various stages of the disease. If such remedy be not known, we must seek for one from among those whose effects resemble the most striking symptom present (*Organon*, par. 232).

In intermittent fever, of a sporadic or epidemic character (not the endemics of marshy districts), the symptoms during the stage of chill, of heat, and of perspiration, with all their conditions and accessories, must be considered, and particularly those of the stage that is the most pronounced; but the symptoms during the apyrexia are of still greater importance, and must carry greater weight in selecting the simillimum (*Organon*, par. 235). The remedy in such cases is generally found among the non-psoric; but in all cases the remedy should be administered only after the paroxysm (*Organon*, par. 236).

If relapses occur, in spite of the administration of the appropriate remedy, an anti-psoric must be given (*Organon*, pars. 240 and 243), and this rule would equally apply to all acute diseases where latent miasmatic symptoms have been suddenly aroused. If the subject of endemic intermittent fever, who leads a regular life, free from excesses, but still inhabiting a marshy district, does not soon recover under the influence of the non-psoric remedy, we may be positive that psora exists, and his cure is only possible through the aid of the anti-psoric remedies (*Organon*, par. 244). In some cases the patient must be removed from the noxious conditions under which he is living.

I cannot close these remarks upon the rules regulating the selection of the appropriate homœopathic remedy without some allusion to Hahnemann's clear and discriminating observations relating to acute epidemics. He tells us to be careful before being satisfied that we have obtained the simillimum for a prevalent epidemic to examine several patients, for seldom do we find in a few cases all the determining symptoms (*Organon*, par. 101). He warns us to distinguish between the symptoms which are almost invariably present from those which are only occasional, and to be guided accordingly in estimating their value. We are, too, precautioned against taking it for granted that one epidemic must necessarily resemble a preceding one, and, consequently, make the mistake of resorting to the same remedies in both cases (*Organon*, par. 102).

I had originally designed to illustrate most of Hahnemann's principles with clinical cases which have come under my observation from time to time, but this monograph gradually assumed such proportions that I have reluctantly to forego this intention. Whether it has lost or gained in interest by this omission I leave to you to decide.

We do not all proceed alike in securing the simillimum. Hahnemann's injunction, as given in his *Materia Medica Pura* (Vol. I, p. 23; Dudgeon's translation), are: "To jot down after each symptom all the medicines which can produce such a symptom with tolerable accuracy," "bearing in mind the circumstances under which they occur, that having a determining influence on our choice, and proceed in the same way with all the other symptoms." "From the list so prepared we shall be able to perceive which among the medicines homœopathically covers the most of the symptoms present, especially the most peculiar and characteristic ones—and this is the remedy sought for, bearing in mind the great importance of the later symptoms." These injunctions are, perhaps, more generally followed than any.

Many physicians, however, adopt Bœnninghausen's plan, which is to consider, first, the moral and intellectual faculties, then locality, sensations, aggravations, and ameliorations and concomitants. This method is best carried out by the use of Allen's *Bœnninghausen's Therapeutic Pocket-Book.* But if a case to be prescribed for reveals no dominating, no characteristic, no peculiar symptom, then we adopt the numerical method, and this is the most readily carried out by using Guernsey's *Bœnninghausen's Repertory,* with which you all are undoubtedly familiar. When we have in this way found the remedy or remedies which correlate with the entire group of symptoms we next consult the *Materia Medica* for our final choice.

I use either one or the other of these two methods, and occasionally Guernsey's Key-Note system, subsequently carefully studying, rubric by rubric, the *Materia Medica,* until I find the simillimum, or the remedy which compares best with the case under consideration. I confess I have always had a strong penchant for working out the simillimum by Guernsey's Key-Note system, which, according to my impression, is merely a promulgation of Hahnemann's idea of characteristic symptoms. Often we are enabled by this plan to choose the remedy very quickly. I incline to the belief that this was the system adopted by those physicians in our school who have earned the reputation of being rapid prescribers.

Hahnemann's three rules are followed by all true homœopathicians. Although two of them have been already alluded to, allow me to sum them up as follows:

1. The choice of a remedy must depend upon the analogy between the symptoms of disease and the symptoms of the drug, and more especially the characteristic ones.

2. The curative remedy must be looked for among those which act in a contrary direction to the symptoms of the disease—*i. e.,* when the symptoms progress from the exterior to the central organs, or from below upwards, the preference must be given to remedies whose action proceeds from the vital organs to the surface of the body, from within outward, or from above downward. Such remedies are found among the antipsorics. "The metaphysics of our science tells us, that all drug diseases (paranosses) are in their essence and offspring, opposite to the whole mass of epidemic, contagious, and other diseases, all of the latter originating from a conflux of causes (synnoses)."

3. The recently developed symptoms are the first to disappear, under successful homœopathic treatment, while the older, more permanent ones, vanish later. The remembrance of this will often prove of the greatest utility to the practitioner, enabling him, on the one hand, to cure with precision, and on the other, to foresee the impossibility of securing a complete cure. If, after the administration of the carefully selected remedy, the symptoms do not yield in the inverse order of their appearance—the later ones vanishing first—we may rest confident that the patient, although he may appear to improve in health, will suffer relapses, sooner or later. Hering, who was one of the greatest lights among the followers of our respected master, added another valuable rule to Hahnemann's three. He repeatedly observed and demonstrated that affections proceeding from one side of the body to the other are more effectively removed by medicines which act in the opposite direction. This might be considered a development of Hahnemann's third rule (*Hahn. Monthly*, Vol. I, p. 49).

Hering also stated that he took it as of good augury to the welfare of the patient if an eruption followed the administration of a remedy; on the other hand, if a pre-existing eruption disappeared, without an improvement in the mental or general symptoms, he considered his prescription faulty and he promptly administered a new remedy.

The above constitutes the principal rules laid down by the truly inspired founder of Homœopathy. No one who has fairly endeavored to put them into practice has failed to meet with successful results in the treatment of the sick—aye, results often bordering upon the marvelous. These rules are based upon the thorough knowledge of the laws relating to science and medicine, and the correlating branches, hygiene, physiology, pathology, and pharmaco-dynamics. How admirably disease and therapeutics are connected and associated, without a flaw in the chain of logic or observation. The theory of the existence of a vital force playing such an important physiological *rôle*, as Hahnemann ascribes to it, may be hypothetical, but there has not been advanced by any medical reformer any more plausible suggestion in explanation of the manifestation of pathological states and their causes. An absolute definition of disease could not be given, and he wisely abstained from it. Mental influence in causing disease is a strong point in favor of Hahnemann's theory, and he very ably alludes to the fact. There is nothing hypothetical in Hahnemann's method. He invents nothing; he simply relates medical facts, founded upon tradition, observation, investigation, and experience. Others before him had recognized a law of similars in medicine, but no one had the genius to found upon it a whole system of medicine based upon eternal, unchanging, natural laws. One rule laid down is the natural and consistent outcome of the other, establishing a perfect accord between and correlating alike disease and therapeutics. Notice how admirably he ever associates cause and effect, and how severe and positive are his rules, and yet so comprehensive. Hahnemann's aim and goal was to remove the diseases besetting humanity, and he proceeded methodically and logically to attain his end, with the result that he has made known to the world the only true law of therapeutics, to the lasting benefit of his race. In truth, it may be said, no greater reformer in the art of medicine, or greater benefactor to humanity has ever lived.

And now, gentlemen, let me say, I am far from satisfied with the result of my labors. It seems to me that I have but imperfectly treated a very weighty matter, but doubtless there are others here who will supply these deficiencies in subsequent essays. And yet, I need hardly say, its preparation has taken up much time and the careful study of all of Hahnemann's works. In spite of all the short-comings of this paper, I do not regret the hours thus spent, for I feel that I have derived no little profit from it. And it is my earnest hope that its perusal may benefit you.

In concluding let me say, we have serious duties to fulfill in our mission as homœopathicians—a duty to the philosophical and beneficent law handed down to us by the immortal master, who bade us maintain, in all its integrity, the grand legacy he bequeathed his followers, and a duty to our patients who entrust us with what is most precious to them—their lives and welfare.

Hahnemann has left us plans for a medical edifice, magnificent and comprehensive, judged by all the canons of art and science; but, of course, some of the details necessary to its completion he did not live long enough to consummate. It remains for us to perfect and complete them, that we may boast in the evening of our lives that we have not lived in vain. Hahnemann truly says: "In a science in which the welfare of mankind is so much concerned, any neglect to make ourselves masters of it becomes a crime." Let us all, then, do our duty faithfully and diligently, fearlessly and with singleness of purpose, till we become better and better equipped to cope with disease and death, and endeavor to sweep away most of the ills besetting poor humanity. There is solace and comfort, too, in the thought that what we do here, may be consummated and made perfect in that life awaiting us, beyond the present sublunary sphere we inhabit.

